# Predicting chronic wasting disease in white-tailed deer at the county scale using machine learning

**DOI:** 10.1038/s41598-024-65002-7

**Published:** 2024-06-22

**Authors:** Md Sohel Ahmed, Brenda J. Hanley, Corey I. Mitchell, Rachel C. Abbott, Nicholas A. Hollingshead, James G. Booth, Joe Guinness, Christopher S. Jennelle, Florian H. Hodel, Carlos Gonzalez-Crespo, Christopher R. Middaugh, Jennifer R. Ballard, Bambi Clemons, Charlie H. Killmaster, Tyler M. Harms, Joe N. Caudell, Kathryn M. Benavidez Westrich, Emily McCallen, Christine Casey, Lindsey M. O’Brien, Jonathan K. Trudeau, Chad Stewart, Michelle Carstensen, William T. McKinley, Kevin P. Hynes, Ashley E. Stevens, Landon A. Miller, Merril Cook, Ryan T. Myers, Jonathan Shaw, Michael J. Tonkovich, James D. Kelly, Daniel M. Grove, Daniel J. Storm, Krysten L. Schuler

**Affiliations:** 1 Wildlife Health Lab, Cornell University, Ithaca, NY USA; 2 Desert Centered Ecology, LLC, Tucson, AZ USA; 3 Department of Statistics and Data Science, Cornell University, Ithaca, NY USA; 4 Minnesota Department of Natural Resources, Nongame Wildlife Program, Saint Paul, MN USA; 5 Department of Fisheries and Wildlife, Michigan State University, East Lansing, MI USA; 6 Center for Animal Disease Modelling and Surveillance, University of California, Davis, CA USA; 7 Arkansas Game and Fish Commission, Little Rock, AR USA; 8 Florida Fish and Wildlife Conservation Commission, Gainesville, FL USA; 9 Georgia Department of Natural Resources, Social Circle, GA USA; 10 Iowa Department of Natural Resources, Ames, IA USA; 11 Indiana Department of Natural Resources, Bloomington, IN USA; 12 Kentucky Department of Fish and Wildlife Resources, Frankfort, KY USA; 13 Maryland Department of Natural Resources, Annapolis, MD USA; 14 Michigan Department of Natural Resources, Grand Rapids, MI USA; 15 Minnesota Department of Natural Resources, Wildlife Health Program, Forest Lake, MN USA; 16 Mississippi Department of Wildlife, Fisheries, and Parks, Jackson, MS USA; 17 New York State Department of Environmental Conservation, Delmar, NY USA; 18 North Carolina Wildlife Resources Commission, Raleigh, NC USA; 19 Ohio Department of Natural Resources, Athens, OH USA; 20 University of Tennessee, Nashville, TN USA; 21 Wisconsin Department of Natural Resources, Madison, WI USA; 22 Present Address: Texas A & M Transportation Institute, Austin, TX USA; 23 Present Address: U.S. Fish and Wildlife Service, Tucson, AZ USA

**Keywords:** Statistics, Ecological epidemiology, Ecological epidemiology, Software

## Abstract

Continued spread of chronic wasting disease (CWD) through wild cervid herds negatively impacts populations, erodes wildlife conservation, drains resource dollars, and challenges wildlife management agencies. Risk factors for CWD have been investigated at state scales, but a regional model to predict locations of new infections can guide increasingly efficient surveillance efforts. We predicted CWD incidence by county using CWD surveillance data depicting white-tailed deer (*Odocoileus virginianus*) in 16 eastern and midwestern US states. We predicted the binary outcome of CWD-status using four machine learning models, utilized five-fold cross-validation and grid search to pinpoint the best model, then compared model predictions against the subsequent year of surveillance data. Cross validation revealed that the Light Boosting Gradient model was the most reliable predictor given the regional data. The predictive model could be helpful for surveillance planning. Predictions of false positives emphasize areas that warrant targeted CWD surveillance because of similar conditions with counties known to harbor CWD. However, disagreements in positives and negatives between the *CWD Prediction Web App* predictions and the on-the-ground surveillance data one year later underscore the need for state wildlife agency professionals to use a layered modeling approach to ensure robust surveillance planning. The *CWD Prediction Web App* is at https://cwd-predict.streamlit.app/.

## Introduction

Chronic wasting disease (CWD) is a transmissible spongiform encephalopathy that infects members of the Cervidae family^1^. The disease stems from the misfolding of prion proteins, leading to neurodegeneration, weight loss, altered behavior, and eventual death^2^. Since first detected in the 1960s, CWD continues to spread through wild and captive cervids across North America^3^. To date, 34 United States (US) state wildlife agencies and four Canadian provincial wildlife agencies have detected CWD in at least one wild cervid herd^3^.

Wildlife agencies in North America have established surveillance programs to detect CWD in wild cervid populations^4^. Such programs focus on identifying locations most likely to harbor CWD and provide the best opportunity to manage the disease while prevalence is low^5^; however, these programs constitute an enormous monetary and human resource cost to agencies^6^. Accordingly, post hoc evaluation of existing surveillance data has focused on pinpointing variables in association with the emergence and spread of CWD to further inform the next year of surveillance^7^.

Anthropogenic factors such as transport and captivity^5,8, 9^ of cervids and natural movements^8^ of cervids can contribute to initial introduction of CWD. Persistence of prions in the environment^10^, soil types^11^, baiting and feeding^12^, forest cover^13^, water^14^, cervid density^15^, and natural movements^8^ contribute to disease spread. Authority for non-imperiled terrestrial wildlife, including most deer species, resides with state and provincial governments^16,17^; as a result, management and surveillance efforts for CWD are highly variable between jurisdictions.

Important and complex questions are driving rapid development, refinement, and use of technology in ecology^18,19^. Among these technologies are machine learning (ML) techniques^20^, which are already revolutionizing analyses in wildlife conservation^21,22^. For example, deep learning has used wildlife imagery to propel detection, inventory, and classification of animals^23^. Full implementation of ML technologies into wildlife science, however, is slowed by our limited ability to rapidly generate high-resolution and standardized data across complex ecologies^24^. Nevertheless, ML is a promising tool for detecting or tracking diseases^25,26^.

A branch of ML is classification, where the goal is to appropriately sort phenomena into categories. Well known classifiers include random forest (RF), decision tree (DT), gradient boosting (GB), and light gradient boosting (LGB) algorithms. A RF is an ensemble of decision trees, where each tree classifies the phenomenon, then votes on the final classification^27^. A DT uses decision rules to divide data further and further into ultimate classifications^28^. The GB is another tree-based ensemble classifier that uses a gradient descent optimization much like binary regression problems^29^. Finally, the LGB functions like GB but with faster computing and improved accuracy^30^.

Statisticians compare ML classifiers using a host of performance summaries. A confusion matrix illustrates the distribution of true negatives (TN), true positives (TP), false negatives (FN), and false positives (FP). Subsequent metrics to assess the performance of ML classifiers use information from the confusion matrix, including accuracy, sensitivity, specificity, precision, recall, F1-score, the receiver-operating-characteristic-area-under-curve (ROC), and area-under-the-curve (AUC)^31,32^.

Our goal was to apply ML classifiers to a regional CWD surveillance dataset to develop a novel model that predicts CWD incidence in wild white-tailed deer (*Odocoileus virginianus*) in counties of 16 states in the midwestern and eastern US. Our objectives were to (1) fit ML classifiers to historical surveillance data, (2) use performance metrics to identify the best classifier, (3) assess which cofactors contribute to the prediction of CWD-status at the county level, and (4) program a user-friendly website application containing the predictive model.

## Results

The Pooled Dataset consisted of 31,636 combinations of counties (1438) and season-years (22), spanning over two decades (1 July 2000–30 June 2022). The Pooled Dataset included variables depicting disease introduction risk (*Cervid_facilities, Taxidermists, Processors, Captive_status*), regulations surrounding disease introduction risk (*Breeding_facilities, Hunting_enclosures, Interstate_import_of_live_cervids*, *Intrastate_movement_of_live_cervids, Whole_carcass_importation*), disease establishment risk (*Buck_harvest*, *Doe_harvest*, *Total_harvest*), environmental variables (*Latitude*, *Longitude*, *Area, Forest_cover, Clay_percent, Streams, Stream_Length*), diagnostic tallies (*Tests_positive*, *Tests_negative*), and regulations surrounding both introduction and establishment risk (*Baiting, Feeding, Urine_lures*). Details of each variable appear in the data readme^33^. Of the 31,636 records, 1.98% (626/31,636) depicted counties with at least one case of CWD in deer (CWD-positive) and 98.02% (31,010/31,636) depicted counties where CWD had not been detected (CWD-non detect).

**Figure 1:**
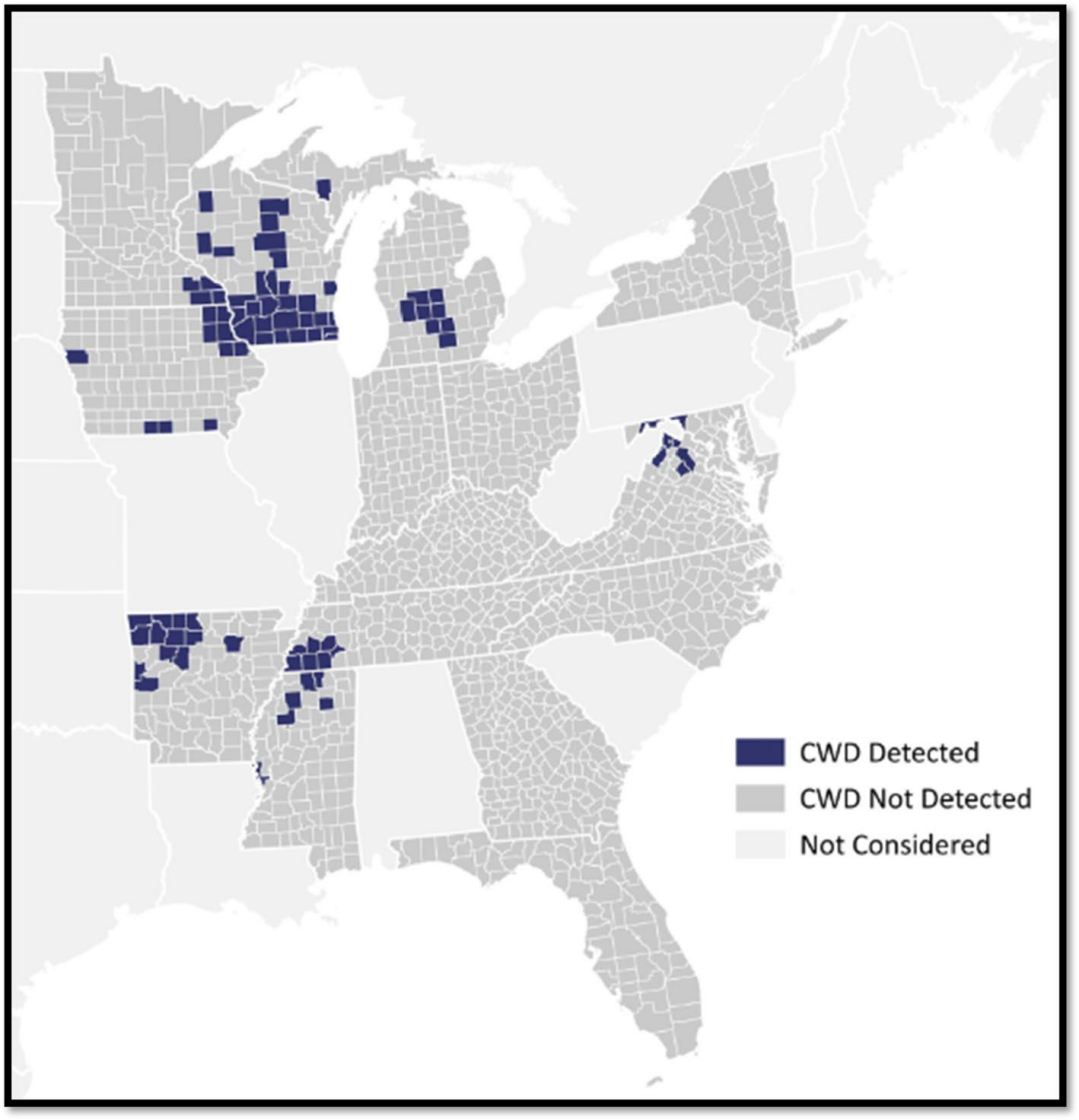
The known status of chronic wasting disease (CWD) in wild white-tailed deer by county in the 2019–20 season according to the results of surveillance testing by US state wildlife agencies^33^. CWD Detected represents counties where governing wildlife officials confirmed at least one CWD-positive case in wild, white-tailed deer in the 2019–20 season. CWD Not Detected represents counties where governing wildlife officials conducted CWD testing in 2019–20 in wild, white-tailed deer, but did not confirm CWD in any subject. Not Considered represents counties that did not exist in the Pooled Dataset^33^. Map was created in QGIS (version 3.32.2-Lima)^60^.

The Orthogonal Dataset consisted of 1438 combinations of counties (1438) and season-years (1) spanning the time period from 1 July 2019–30 June 2020. The Orthogonal Dataset included variables depicting disease introduction risk (*Cervid_Facilities, Captive_status*), regulations surrounding introduction risk (*Hunting_enclosures, Whole_carcass_importation*), disease establishment risk (*Total_harvest*), environmental variables (*Forest_cover, Clay_percent, Streams*), and regulations surrounding both introduction risk and establishment risk (*Baiting, Feeding, Urine_lures*). Details of each variable appear in Table 1. Of the 1,438 records, 5.91% (85/1438) depicted CWD-positive counties, 94.09% (1353/1438) depicted CWD-non detect counties (Fig. 1).

**Table 1:** Definitions of variables in the Orthogonal Dataset, borrowed with permission^33^.

Details	Variable name	Definition
Positive Number 0 $\le$ x $\le \infty$	*Cervid_Facilities*	Number of privately owned premises in the management area that maintain cervids in captivity for the purposes of breeding, farming, display, and/or enclosed (fenced) hunting operations known to the wildlife agency in the given season-year or in the most recent prior season-year for which the number is known^33^
	*Total_harvest*	Number of wild white-tailed deer harvested by hunters in the management area in the season-year or in the most recent prior season-year for which the number is known and reported to the wildlife agency^33^
	*Streams*	Average distance in meters from any location within a management area to the nearest high order stream (Strahler order 4 or greater) as defined in the National Hydrography Dataset (NHD) Plus; derived by intersecting the NHD Plus and USCB Cartographic Boundary File^33^
Proportion 0 $\le$ x $\le$ 1 Where 0 indicates no forest, and 1 indicates all forest	*Forest_cover*	Proportion of the area (including inland waters) classified as forest in the North American Land Change Monitoring System^33^. Land cover classes included as “forest” include: class 1 (temperate or sub-polar needleleaf forest), class 2 (sub-polar taiga needleleaf forest), class 3 (tropical or sub-tropical broadleaf evergreen forest), class 4 (tropical or sub-tropical broadleaf deciduous forest), class 5 (temperate or sub-polar broadleaf deciduous forest), class 6 (mixed forest), class 7 (tropical or sub-tropical shrubland), class 8 (temperate or sub-polar shrubland), and class 14 (wetland)^33^
Percentage 0 $\le$ x $\le$ 100 Where 0 indicates no clay, and 100 indicates all clay	*Clay_percent*	Average percentage of clay in the upper 50 cm of soil, as calculated using the clay content at standard depths^33^
Ordinal x $\in$ {0, 0.5, 1} 0: Governing officials restrict this activity 0.5: Governing officials partially restrict this activity 1: Governing officials allow this activity	*Hunting_enclosures*	Indicates whether captive cervid hunting is allowed in the administrative area^33^
	*Feeding*	Indicates whether feeding of cervids, defined as the placement of substances, including grains, minerals, hay, or other food materials, used to attract cervids for non-hunting purposes is allowed in the administrative area^33^
	*Whole_carcass_importation*	Indicates whether the administrative area allows importation of whole cervid carcasses from other administrative areas^33^
	*Baiting*	Indicates whether baiting of cervids, defined as the placement of substances, including grains, minerals, hay, or other food materials, used to attract cervids for the purpose of hunting is allowed in the administrative area^33^
	*Urine_lures*	Indicates whether the use of urine lures, defined as natural or synthetic urine-based attractants for hunting purposes, is allowed in the administrative area^33^
Binary x $\in$ {0, 1} 0: CWD has never been confirmed 1: CWD has been confirmed	*Captive_status*	Indicates whether CWD has been detected at one or more captive cervid facilities in the management area in the given season-year or in any prior season-year and reported to the USDA APHIS^33^

Definitions of variables in the Pooled Dataset are in the Supplement.

The Balanced Orthogonal Dataset consisted of a subset of 158 counties depicting conditions in the 2019–20 season-year. Of the 158 counties, 50% (79/158) represented CWD-positive counties and 50% (79/158) represented randomly selected CWD-non detect counties. All counties in the Balanced Orthogonal Dataset contained values for hunter harvest (although that value could have been zero). [Note that of the 85 total positive counties available in the Orthogonal Dataset, six counties in the US state of Mississippi were excluded from the Balanced Dataset due to missing *Total_harvest* values.] The Training Dataset consisted of 126 (80%) records randomly selected from the Balanced Orthogonal Dataset while the Testing Dataset consisted of the remaining 32 (20%) records of the Balanced Orthogonal Dataset. Summary statistics for each variable in the Pooled, Orthogonal, Balanced Orthogonal, Training, and Testing Datasets are provided in the Supplement.

**Figure 2:**
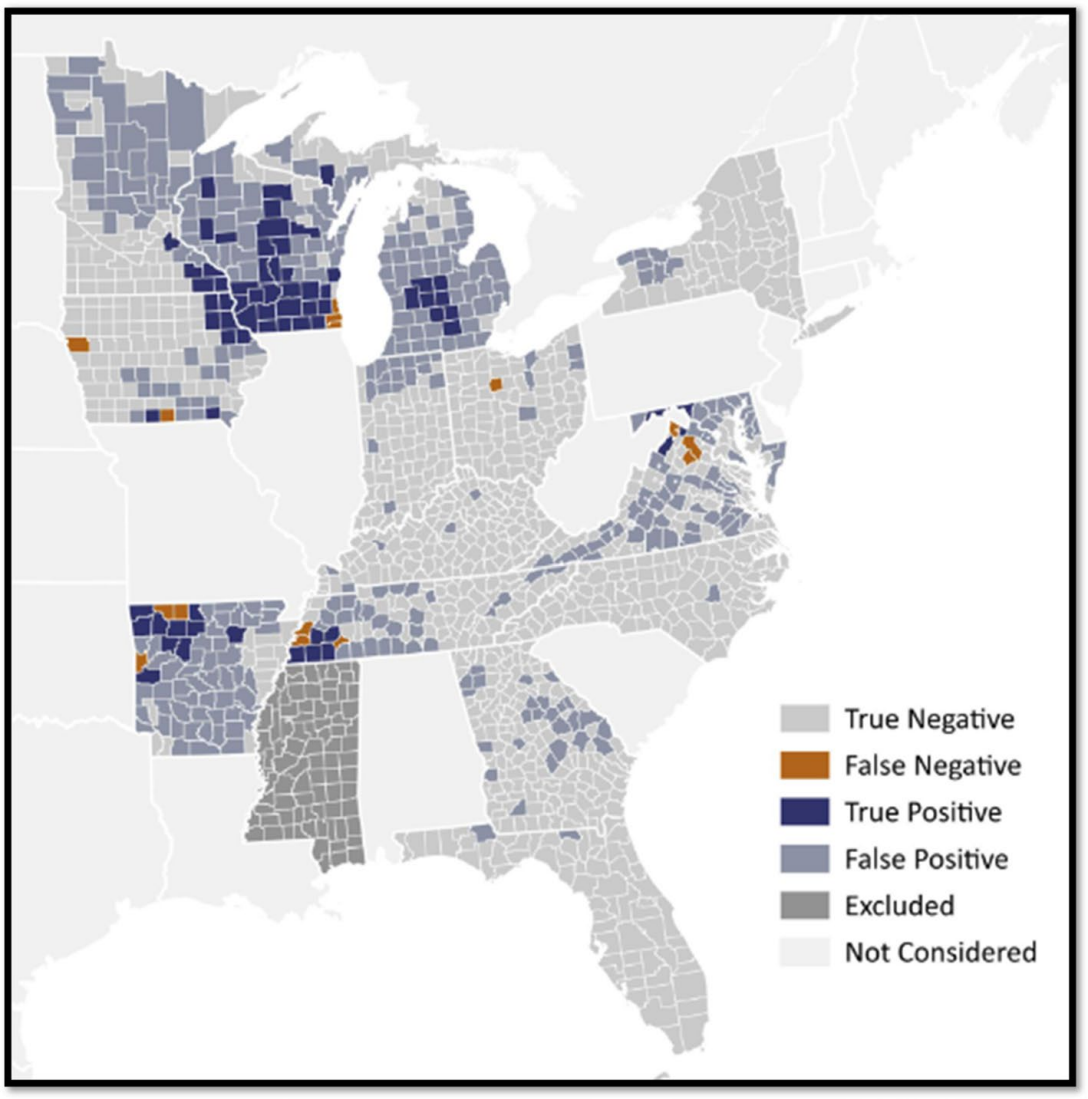
Comparison of chronic wasting disease (CWD) status in free-ranging white-tailed deer in season-year 2020–21 between the *CWD Prediction Web App* and state surveillance data^33^. True Negatives (TNs) occurred when the *CWD Prediction Web App* prediction and the surveillance data agreed that CWD-status was CWD-non detect for the county in the season-year 2020–21. True Positives (TPs) occurred when the *CWD Prediction Web App* prediction and the surveillance data agreed that CWD-status was CWD-positive for the county in the season-year 2020–21. False Negatives (FNs) occurred when the *CWD Prediction Web App* predicted CWD-non detect, but the surveillance data declared CWD-positive for the county in season-year 2020–21. False Positives (FPs) occurred when the *CWD Prediction Web App* predicted CWD-positive, but the surveillance data declared CWD-non detect for the county in season-year 2020–21. Excluded represents counties omitted from predictions because harvest data was either not collected or could not be approximated by-county. Not Considered represents areas omitted from the Pooled Dataset^33^. Two sources of known error can cause predictions to deviate from reality: (1) model classification error and/or (2) error in CWD-status from surveillance. Specific to Minnesota, a third known error could cause predictions to deviate from reality: (3) error arising from the conversion of harvest data collected in Deer Permit Areas into county-approximations (see the Supplement for specific details). Map was created in QGIS (version 3.32.2-Lima)^60^.

The Balanced Orthogonal Dataset set contained non-linear data and outliers, so we analyzed the Training and Testing Datasets using four supervised ML algorithms: Random Forest (RF), Decision Tree (DT), Gradient Boosting (GB), and Light Gradient Boosting (LGB)^27–30^. We used k-fold validation to determine the best hyperparameters and avoid overfitting the model (see Supplement). We found that the LGB was the best model among those evaluated due to its balance between training and testing performance across multiple validations. While RF and GB initially appeared strong due to high training performance and testing performance, the overfitting concern diminished their appeal when compared to LGB, which demonstrated a more balanced performance and superior capacity for generalization. Light Gradient Boosting achieved correct classification of CWD-positive counties in 71.88% of the records in the Testing Dataset (with the highest average accuracy across the fivefold validation of 76.25%; see Supplement). Similarly, the LGB achieved a F1-score of 68.75%, precision of 73.33%, recall of 64.71%, and ROC of 78.82%, implying strong consistency, specificity, sensitivity, and discriminative power (see Supplement). Due to its superior performance in the cross validation, we deemed the LGB to be the strongest performer in predicting the status of CWD at the county level in the midwestern and eastern US given these data. Further assessment of the LGB model revealed that the most influential variables included in the model for these predictions of CWD (Fig. 2) included regulations surrounding risk of anthropogenic introduction of infectious materials (use of *urine lures* and importation of *whole carcasses*) and natural deer movement to reach water (distance to *streams*; see Supplement).

Investigation of the LGB model revealed good accuracy when we compared *CWD Prediction Web App* predictions to the results of the field-based surveillance from the subsequent year (i.e., the season-year 2020–21). Relative to the CWD-status from on-the-ground surveillance in 2020–21, the *CWD Prediction Web App* predictions contained 75% accuracy, 82% sensitivity, 74% specificity, 29% F1-score, 82% recall, and 78% ROC. The *CWD Prediction Web App* showed 946 TNs, 70 TPs, 15 FNs, and 325 FPs relative to known data from the 2020–21 season-year (Table 2; Fig. 2).

**Table 2:** The confusion matrix of the best Light Gradient Boosting (LGB) model when *CWD Prediction Web App* predictions were compared against on-the-ground surveillance in white-tailed deer in the season-year 2020–21.

		Model predictions	
		CWD-non detect	CWD-positive
On-the-ground surveillance	CWD-non detect	True negative	False positive
		70% (946/1,356)	24% (325/1,356)
	CWD-positive	False negative	True positive
		1% (15/1,356)	5% (70/1,356)

The *CWD Prediction Web App* had 70 TPs for the 2020–21 season-year, 66 of which constituted counties already known to be CWD-positive in white-tailed deer from the 2019–20 surveillance data. The remaining four TPs depicted counties that indeed turned positive in white-tailed deer for the first time in the 2020–21 season-year, just as the model predicted (Dakota county, Minnesota; Shawano, Washington, and Wood counties, Wisconsin). The *CWD Prediction Web App* had 325 FPs relative to surveillance data from the 2020–21 season-year.

The *CWD Prediction Web App* had 946 TNs for the 2020–21 season-year. The *CWD Prediction Web App* had 15 FNs for the 2020–21 season-year, 13 of which were counties the *CWD Prediction Web App* knew were positive from the 2019–20 but incorrectly assigned to be negative in the 2020–21 season-year. The remaining two counties (Wyandot county, Ohio; Lauderdale county, Tennessee) were negative in 2019–20 and detected a positive in 2020–21, but the *CWD Prediction Web App* did not successfully predict that transition in CWD-status. The *CWD Prediction Web App* is at https://cwd-predict.streamlit.app/. The code is available at https://github.com/sohel10/lgbm.

## Discussion

Despite the governing autonomy of management agencies, free-ranging wildlife spans jurisdictional boundaries. Consequently, wildlife agencies across North America would benefit from cooperative efforts designed to understand shared risk factors of disease. Our study was the first to use regional data that represent a single species exposed to diverse management goals, herd dynamics, habitat types, and regulations spanning 16 US states. As well, our cutting-edge application of ML techniques to wildlife health data enabled us to identify counties that contain characteristics similar to counties around the midwestern and eastern US with confirmed CWD.

Our results from the LGB algorithm revealed that regulations have a bearing on the CWD predictions shown in Fig. 2. Indeed, wildlife professionals have long pointed to risk factors for CWD introduction from human-assisted movement of prions via live cervids, carcasses, trophy heads, deer parts, and urine lures^8,9^. Consequently, wildlife agencies have installed a variety of regulatory measures to limit or extinguish avenues for introduction from anthropogenic sources^34^. Our results from the LGB algorithm further corroborates prior knowledge that natural movements of deer^35^ (here specifically to visit water sources) is an important feature driving the predictions of CWD-status. However, we strongly caution that these features and their importances may be phenomena of the data and not absolute. Afterall, the other three candidate algorithms performed similarly with these data (see Table S2 in the Supplement), and their results hinged on entirely different sets and ranks of factor importances. Specifically, the RF algorithm ranked hunter harvest (a proxy for deer density)^36^, clay-based soils^37–41^, forest cover^13^, and then distance to streams^35^ as the most important features driving its predictions of CWD-status, in that order. The DT algorithm ranked hunter harvest^36^, distance to streams^35^, clay-based soils^37–41^ and then forest cover^13^ as the top features of importance driving its predictions of CWD-status, in that order. And finally, the GB algorithm ranked hunter harvest^36^, distance to streams^35^, forest cover^13^, and then clay-based soils^37–41^ as the top features driving its predictions of CWD-status, in that order. With every algorithm, there is some way to corroborate the importances using prior research. These seemingly similar results beg the question: if accuracy was similar across LGB, RF, DT, and GB algorithms, then how did we pick the LGB algorithm to present in Fig. 2? The answer lies in the underlying mathematics: we recognized that we do not yet have enough data for the obvious superior predictor to emerge, so we chose the predictor with the highest current *average accuracy* (even when other algorithms outperformed LGB by random chance in any given singular instance). As well, the LGB demonstrated a more optimal balance between training and testing accuracy than the RF, DT, and GB options. As more data are incorporated into the future fitting of these ML models (see additional discussion below), performance *averages* will settle into the asymptotic means according to the Law of Large Numbers^42^, and any of these four algorithms (with their corresponding feature importances and ranks) could emerge as the superior predictor of CWD-status.

Factor importances from the LGB, RF, DT, and GB algorithms arose from a spatially diverse dataset, and therefore, results offer additional insights relative to those obtained using more localized data. However, these factors emerged as important to these algorithms only out of the factors assessed, and other factors that may be intrinsic pathobiological properties of CWD were omitted from this study. For example, the Pooled Dataset^33^ did not contain data on potentially relevant drivers of CWD-status like weather, prion strain, diagnostic test type, deer genetics, explicit dispersal of deer^35^, management strategies^8^, existence of sympatric susceptible species^43^, illegal activities such as unapproved movement or release of captive cervids from CWD-positive herds^44,45^, or geographical proximity to infections in neighboring areas^5^.

Results from the LGB algorithm applied to the Pooled Dataset^33^ revealed that regulations matter in predicting CWD-status. However, the two specific regulations pinpointed by the LGB model (*urine lures* and *whole carcass import*) are confounded with the other regulations that we removed due to high correlation (*breeding facilities*, *interstate import of live cervids*, *intrastate movement of live cervids*). Due to covarying regulations (and our selection procedure regarding the variable to remove and the variable to retain, see methods), rather than taking variable names at face value, we recommend interpreting the importance of urine lures and whole carcasses regulations as proxy for regulations depicting *general human activities that could introduce contamination into the reservoir*.

There are numerous potential improvements to this model. The North American Model of Wildlife Conservation recognizes science as the appropriate tool for directing wildlife resource management^46^; however, it is within the purview of state wildlife agencies to determine the scientific methods that best meet their needs^16,17^. Thus, the first challenge of this work was to find the spatial unit that was the ‘least common denominator’ across all states. Because many agencies represented in the Pooled Dataset^33^ recorded county in their CWD testing (surveillance) data (and ancillary spatial data was collected at a unit such that we could confidently infer county from their reported locations), we elected to conduct our analysis at the county-scale. However, we acknowledge county may not be ecologically relevant to either the biology of cervid herds or the spatial unit of interest to wildlife managers. In addition, our selection of county presented problems for predictions in Minnesota (see discussion below). Nevertheless, there were several advantages to using county. First, the decision enabled us to leverage the power in the largest set of existing CWD surveillance data to create the first-ever regional model depicting predictions of CWD-status in North America. Second, the decision enabled us to compare CWD-status across myriad local configurations (i.e., management and policies) to pinpoint potential intrinsic properties of CWD. While there remains work to pinpoint the best algorithm for predicting CWD-status in North America, our results thus far suggest that regulations, hunter harvest (as a proxy for deer density), and habitat variables (forest, clay, and distance to streams) may play a role in CWD-status regardless of local management decisions and policies. Finally, county is the scale of interest to public health departments^47^ who share interest in tracking CWD in wild herds. The ML method requires a single year of pooled data to train the model and the next year of pooled data to assess predictions. Accordingly, if other scales are of interest in surveillance planning, we suggest that agencies coordinate to collect information at the scale of interest for two consecutive years.

Disagreements in CWD-status between the *CWD Prediction Web App* predictions and surveillance data of the 2020–21 season-year are explainable for all participating states in one of two ways: (Case 1) the *CWD Prediction Web App* predicted CWD-positive, the surveillance data reported CWD-non detect, and CWD truly did not exist in white-tailed deer in that county (and therefore the error was on the part of the model) and (Case 2) the *CWD Prediction Web App* predicted CWD-positive, the surveillance data reported CWD-non detect, but CWD truly existed in white-tailed deer in that county (and therefore the error was on the part of surveillance data). Disagreements specific to Minnesota are explainable in a third known way: (Case 3) the *CWD Prediction Web App* predicted CWD-positive or CWD-non detect status for each county in Minnesota using harvest estimates that themselves deviated from reality. [Despite the lack of information to confidently convert harvest data across spatial scales in Minnesota, proportional allocation was used^33^ to make county-based approximations of harvest from harvest tallies by Deer Permit Areas (DPAs). Sensitivity analysis of *CWD Prediction Web App* predictions relative to alterations in harvest revealed vulnerabilities in binary predictions. Specifically, 100% (52/52) of the predicted CWD-non detect counties and 94.3% (33/35) of the predicted CWD-positive counties in Minnesota hinged on the value of harvest obtained through the county-approximation. There is no way to know if or to what extent county approximations differ from reality. Nevertheless, the Supplement contains the county approximation value of hunter harvest used in predictions as well as the bifurcation point differentiating a CWD-positive prediction from a CWD-non detect prediction for each county in Minnesota.] Error reduction in (Case 1) is attainable by rerunning the model for a single season-year containing all the counties herein plus counties from additional states that have both CWD-positive and CWD-non detect herds (the model cannot be improved by adding additional years of data from counties in states already depicted and cannot be improved by adding counties from new states that do not have CWD). Error reduction in (Case 2) is attainable by ensuring that sufficient samples are taken in each county to be 95% confident that CWD-non detect counties in the data are indeed free-from-disease^48^. Error reduction in (Case 3) case is attainable by pooling regional records with outright comparable units (or spatial scales) or using only records containing sufficient information for one-to-one transformations between units (or spatial scales).

Despite a large dataset and powerful modeling tools, the data underlying the *CWD Prediction Web App* are wrought with statistical and ecological complications. For instance, the Pooled Dataset^33^ reported presence and absence of CWD in a county directly from sample testing data, but did not account for sampling effort, latent introduction time, deer population growth rates, disease transmission, or detection probability^49^. While the Pooled Dataset^33^ constituted the best available regional information regarding CWD-status by county/season-year, we acknowledge that counties deemed to be CWD-free may consist of too few samples to support such a declaration. Should this analysis be repeated with more agency partners, which we recommend, we suggest using data from counties for which there were sufficient samples taken to ensure statistical confidence in the CWD-status. As well, there exists standardized diagnostics for CWD in captive cervid herds^50^, but similar standards do not exist for wild cervids and CWD designation is made by state wildlife authorities. We further suggest the adoption of standardized terminology and definitions surrounding all CWD topics to facilitate comparability of data in future regional studies.

The *CWD Prediction Web App* constitutes an important new tool for CWD surveillance planning, especially when managers overseeing vast areas do not know where to begin testing for the disease. However, we caution the use of the *CWD Prediction Web App* in three ways. First, it might be tempting to use this tool to predict CWD-status in geographical areas smaller than counties, such as Game Management Units. We do not recommend this use until the model underlying the *CWD Prediction Web App* is validated using a known dataset containing true positives and negatives at this geographical scale. Instead, we currently recommend using the Habitat Risk model^51^ for such analyses, should the surveillance data in the area of interest have exact geographical locations. Second, due in part to our findings regarding FNs, the *Web App* should not be used in isolation to determine a sampling strategy nor to replace the collection and testing of tissues conducted by agencies each year in the field. And third, due to our findings of similar predictive performance yet differing feature importances among the four ML algorithms, we do not recommend interpreting the LGB feature importances as absolute truth in CWD-predictions.

The Pooled Dataset^33^ did not contain data on distance to infection, yet the regional map revealed that many predictions of CWD-positive status are largely contiguous to known infections (Fig. 2). While agencies may already be searching for CWD in areas contiguous to core infections, the *CWD Prediction Web App* may be particularly helpful in illuminating counties vulnerable to CWD in non-obvious places. In noncontiguous counties predicted by the *CWD Prediction Web App* to be CWD-positive, we suggest using the *CWD Prediction Web App* in conjunction with other models that pinpoint conditions for in situ outbreaks^7,51, 52^ for surveillance planning. In addition to the error reductions recommended above, we recommend that future ML models better characterize the spread of disease across the landscape by incorporating geographical proximity data or information from diffusion models^53^ which we did not do.

## Conclusion

The *CWD Prediction Web App* produced 325 FPs relative to the subsequent season-year of surveillance. Ostensibly, this may appear to be too much inaccuracy. However, these FPs are quite helpful in understanding regional patterns and vulnerabilities to change in CWD-status. Specifically, the preponderance of FPs signals the counties that warrant increased CWD surveillance in upcoming years, as they share conditions with counties around the region known to harbor CWD. Alternatively, the *CWD Prediction Web App* should not be used in isolation for surveillance planning because it produced 15 FNs relative to the subsequent season-year of surveillance data. Hence, we recommend using the *CWD Prediction Web App* in conjunction with other models to ensure surveillance does not miss introduction in assumed ‘low-risk’ counties. Indeed, a true measure of the accuracy of the *CWD Prediction Web App* will emerge as predictions are followed through time.

This research simultaneously demonstrates the opportunity and limitations of integrating ML into disease surveillance planning. While the first of its kind to rely on such a large initial dataset (31,636 records), by the time we transformed these data for use in the ML algorithms, usable records had diminished to ‘small data’^54^ (158 records). Despite this limitation, we illustrated that it is still possible to build a predictive ML system to predict CWD occurrence across a vast geographical region. We recommend iterative improvements to this model through the inclusion of additional data as ML processes are recursive and responsive to added information. Continued enhancement of the *CWD Prediction Web App* via incorporation of additional data will hone predictions, improve surveillance, and reduce costs for all.

## Methods

We used CWD surveillance and ancillary data from the midwestern and eastern US^33^. Here we refer to this data as the Pooled Dataset. The Pooled Dataset contains multivariate records in white-tailed deer from the US states of Arkansas, Florida, Georgia, Indiana, Iowa, Kentucky, Maryland, Michigan, Minnesota, Mississippi, New York, North Carolina, Ohio, Tennessee, Virginia, and Wisconsin, and spans the season-years 2000–01 to 2021–22^33^. Definitions for each variable appear in the data documentation^33^. Minnesota collected harvest data at the Deer Permit Area (DPA) spatial scale, so proportional allocation was used to convert their recorded harvest data into county-scale approximations^33^.

We checked all variable pairs for multicollinearity and high correlation, then removed one of the offending variables with correlation exceeding 0.7^55^. When applicable, we weighed which variable to remove based on the total number of missing values or if one variable had a higher difficulty of collection in on-the-ground efforts. We removed linearly inseparable data^56^ by retaining only records for the 2019–20 season-year. We chose the 2019–20 season-year, because it was the period for which we had complete data for the largest number of unique counties. We called this subset of the Pooled Dataset the Orthogonal Dataset.

We deemed our response variable in the Orthogonal Dataset to be whether or not the source agency reported at least one wild deer to be CWD-positive in the county during the 2019–20 season-year (i.e., the *Management_area_positive* variable). Imbalances in the binary outcomes (1 means the county is CWD-positive and 0 means the county is CWD-non detect) are known to skew predictions and introduce inaccuracies due to insufficient information about the minority class^57^. We therefore checked for an imbalance in the number of CWD-positive and CWD-non detect counties in *Management_area_positive*, and if present, applied resampling techniques for the majority class (CWD-non detect) to balance the number of CWD-positive and CWD-non detect counties. We created the Balanced Orthogonal Dataset by taking all full records of CWD-positive counties and adding them to the same number of randomly selected CWD-non detect counties. We instructed the computer to randomly partition the Balanced Orthogonal Dataset into two subsets: a Training Dataset comprising 80% of the records [regardless of CWD-status] and a Testing Dataset with the remaining 20% of the records.

We built four ML models to predict the binary outcome of CWD in a county^58^. We selected candidate ML algorithm(s) that aligned with the dataset's characteristics. We used the Training Dataset to create a prediction classifier, then the Testing Dataset to assess the model’s performance in predicting the presence of CWD. We used k-fold cross-validation accuracy to select the hyperparameters of each model^56^.

We used the sci-kit-learn (version 1.4.2)^57^ to assess the performance of each classifier by considering accuracy, F1-score, precision, recall, and ROC simultaneously. We chose the model that demonstrated the best balance between training and testing data, then used the predictor gain method^59^ to evaluate the importance of variables contained in the model. We generated its confusion matrix relative to the subsequent season-year (2020–21) of surveillance data. We programmed the top model into the *CWD Prediction Web App* to predict CWD-status in each county.

### Supplementary Information

Supplementary Information.

## Data Availability

The data are publicly available at 10.7298/7txw-2681.2. The CWD Prediction Web App is at https://cwd-predict.streamlit.app/.
