# Supplementary material for: Predicting chronic wasting disease in white-tailed deer at the county scale using machine learning
Source: Sci Rep. 2024 Jun 22;14:14373. doi: 10.1038/s41598-024-65002-7 (PMC11193737; doi:10.1038/s41598-024-65002-7)
Supplement: Supplementary file 1 — Supplementary Information. [file 41598_2024_65002_MOESM1_ESM.docx]

***Supplement to*:**

**Predicting chronic wasting disease in white-tailed deer at the county scale using machine learning**

**Bias assessment arising from proportional allocation of hunter harvest data**

Minnesota collects harvest data at the Deer Permit Area (DPA) and does not report their harvest data at the county spatial scale. In order to pool their data in with the other US states for this analysis, the authors of the data^29^ needed to convert their harvest data into county scale. Please see the data^29^ for details of that conversion. What appears below is a summary of how that conversion could have propagated through the machine learning algorithm to affect downstream predictions of CWD-status.

*Methods*

For each county in Minnesota, we used the *CWD Prediction Web App* in conjunction with county covariates to find the exact number of deer that needed to be harvested before the *CWD Prediction Web App* predictions flipped from pos-> neg (or from neg-> pos) for that county. We collected the results in Table S4.

*Results*

The *CWD Prediction Web App* predicted 52 counties to be CWD-non detect. All 52 (100%) flipped to positive when harvest increased by a county-specific number of deer, meaning that error in the county-approximation could have driven predictive outcomes (see Table S4).

The *CWD Prediction Web App* predicted 35 counties to be CWD-positive. Of those, 2 (5.7%) remained positive regardless of harvest values, meaning that error in the approximation made no difference in ultimate predictions. The remaining 33 (94.3%) flipped to negative when harvest decreased by county-specific numbers of deer, meaning that error in the county-approximation could have driven predictive outcomes (see Table S4).

*Conclusion*

The outcome of as many as 100% (52/52) predicted CWD-non detects and 94.3% (33/35) predicted CWD-positives hinged on values of harvest in the county-approximation as opposed to (unknown) reality.


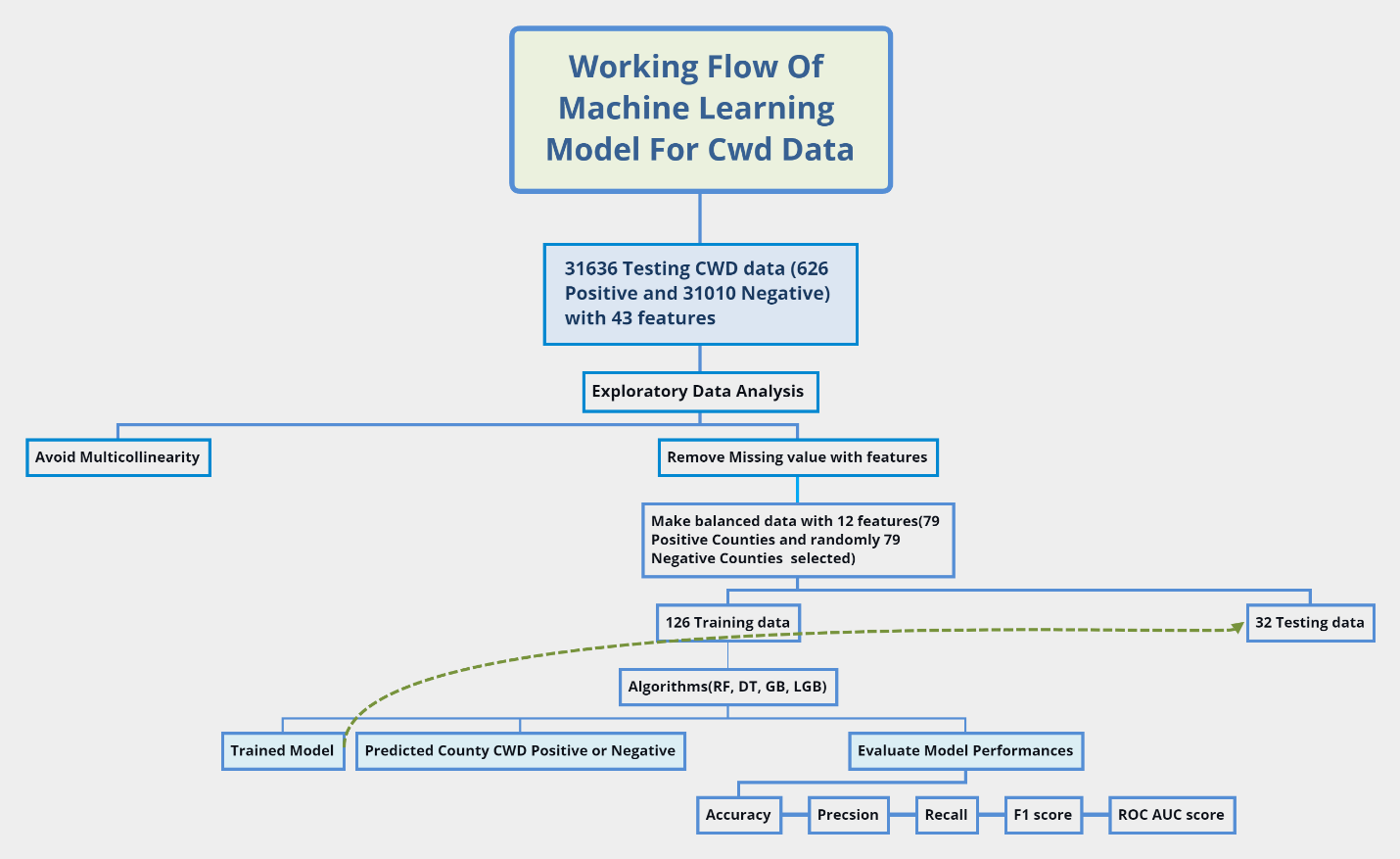
Figure S1. Flow chart of the machine learning methods involved in predicting binary classifications of chronic wasting disease (CWD) in wild white-tailed deer at the county scale.


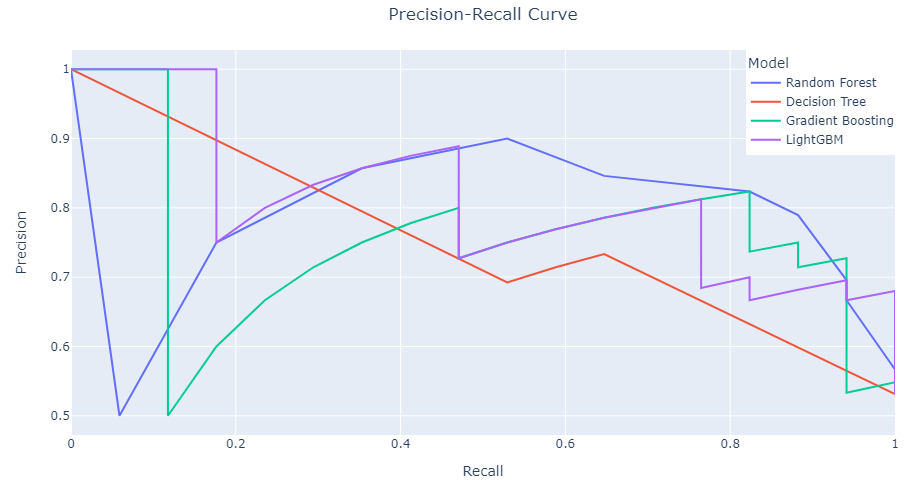


Figure S2. Precision-recall curves of the performance in predicting binary classifications of chronic wasting disease (CWD) in wild white-tailed deer at the county scale. Algorithms include Random Forest, Decision Tree, Gradient Boosting, and Light Gradient Boosting models. Recall (x-axis) represents the ability of the algorithm to identify true positives. Precision (y-axis represents) represents the correctness of positive predictions relative to the known positive status for each county in season-year 2019-20^29^.


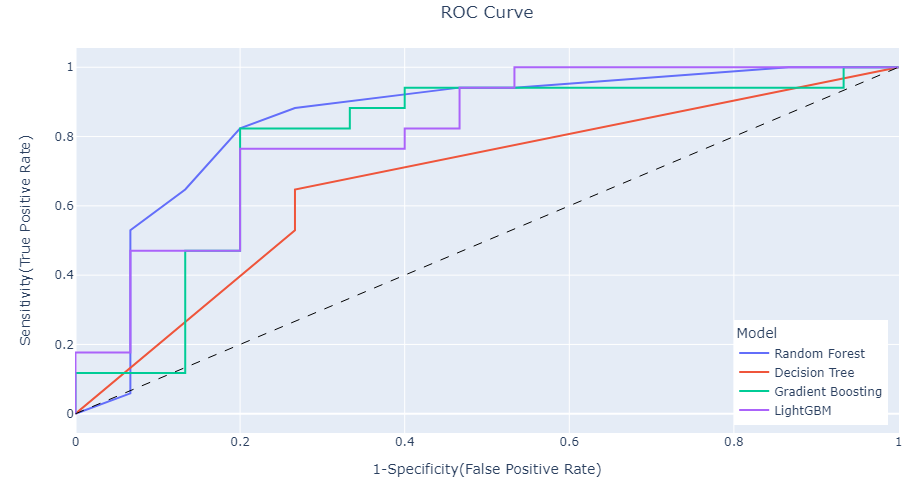


Figure S3. Receiver operating characteristics curves of the performances in predicting binary classification of chronic wasting disease (CWD) in wild white-tailed deer at the county scale. Algorithms include Random Forest, Decision Tree, Gradient Boosting, and Light Gradient Boosting models. The False Positive Rate (x-axis) represents the proportion of times the algorithm incorrectly predicted a CWD-positive relative to the known positive status for each county in season-year 2019-20^29^. True Positive Rate (y-axis) represents the proportion of times the algorithm correctly predicted a CWD-positive relative to the known positive status for each county in season-year 2019-20^29^.


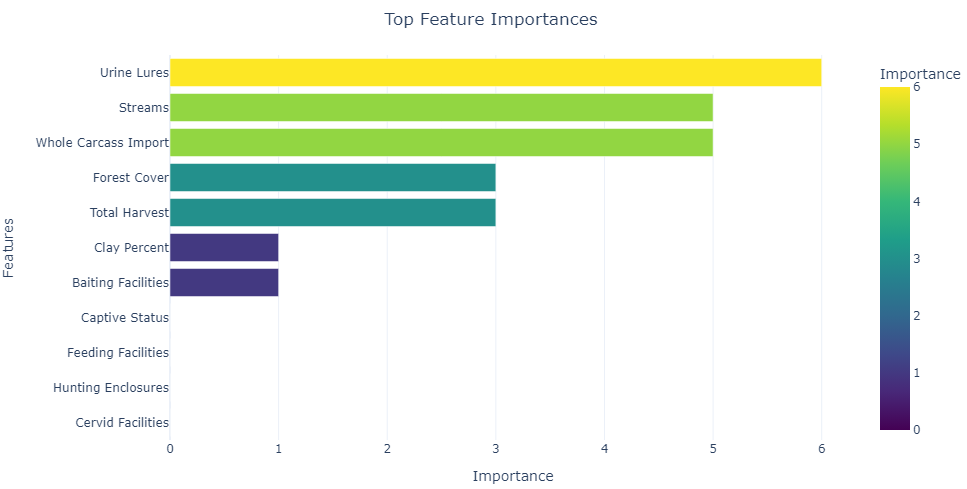


Figure S4. Feature importance plot for the Light Gradient Boosting algorithm when predicting binary status of chronic wasting disease (CWD) in wild white-tailed deer at the county scale. The plot shows that regulations surrounding urine lures (a proxy for deposition of potentially infectious materials into the reservoir) is the most important feature in these predictions of CWD-presence or CWD-non detect at the county scale. Distance to streams (a proxy for deer movement) was next important feature in these predictions, followed by regulations surrounding the importation of whole carcasses (proxy for anthropogenic movement of potentially infectious materials across state lines).


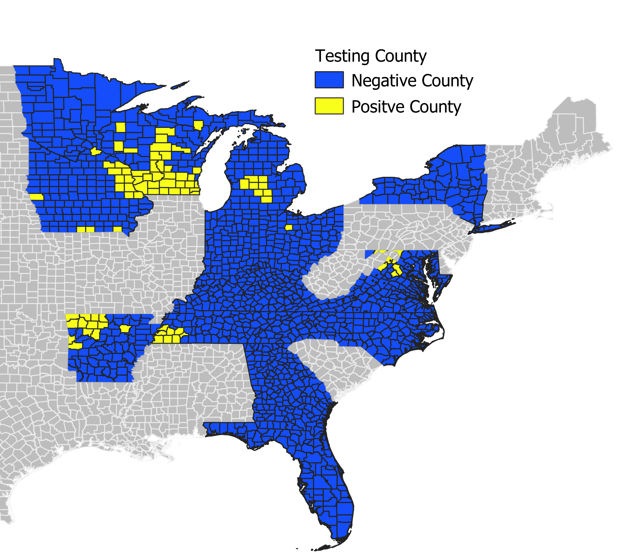


Figure S5. The known status of chronic wasting disease (CWD) in wild white-tailed deer by county in the 2020-21 season according to results of surveillance testing by state wildlife agencies^29^. Yellow areas represent counties where governing wildlife officials confirmed at least one CWD-positive case in wild, white-tailed deer. Blue areas represent counties where governing wildlife officials conducted CWD testing in wild, white-tailed deer, but did not confirm CWD in any subject. Grey areas were not included in the model. Maps were created in the QGIS (version 3.22.3)

Table S1. Variables and dimensions of the Pooled, Orthogonal, Balanced Orthogonal, Training, and Testing Datasets. The abbreviation ‘n’ indicates the number of cells containing data. Values are depicted as mean plus or minus (±) 1 standard deviation, rounded to the nearest tenth.

| Variable | | Dataset | | | | |
| --- | --- | --- | --- | --- | --- | --- |
| Type | Name | Pooled  (31,636 $\times$ 43) | Orthogonal  (1,438$\times$ 12) | Balanced Orthogonal  (158 $\times$ 12) | Training  (126 $\times$ 11) | Testing  (32 $\times$ 11) |
| Positive Number  0 $\leq$ x $\leq$ $\infty$ | Cervid_  facilities | (n = 28,760)  1.17 ± 0.02 | (n = 1,438)  0.70 ± 0.05 | (n = 158)  0.66 ± 0.12 | (n = 126)  0.73 ± 0.14 | (n = 32)  0.41 ± 0.17 |
|  | Taxidermists | (n = 2,833)  6.58 ± 0.12 | Not included | | | |
|  | Processors | (n = 2,187)  2.65 ± 0.06 | Not included | | | |
|  | Total_  harvest | (n = 10,499)  2,326.38 ± 23.18 | (n =1,355)  1,937.86 ±  47.19 | (n = 158)  2,558.77 ± 158.00 | (n = 126)  2,577.77 ± 180.87 | (n = 32)  2,483.97 ± 323.45 |
|  | Area | (n = 31,636)  1,409,268,887 ± 5,277,385 | Not included | | | |
|  | Streams | (n = 31,636)  5,963.78 ± 21.31 | (n = 1,438)  5,963.78 ± 123.46 | (n = 158)  5,450.47 ± 302.85 | (n = 126)  5,444.44 ± 337.89 | (n = 32) 5,474.21 ± 693.39 |
|  | Stream_  Length | (n = 30,602)  118,531.20 ±  496.13 | Not included | | | |
| Proportion  0 $\leq$ x $\leq$ 1 | Forest_  cover | (n = 17,256)  0.49 ± 0.00 | (n = 1,438)  0.49 ± 0.01 | (n = 158)  0.47 ± 0.02 | (n = 126)  0.47 ± 0.02 | (n = 32)  0.45 ± 0.05 |
| Percentage  0 $\leq$ x $\leq$ 100 | Clay_  percent | (n = 31,636)  21.10 ± 0.03 | (n = 1,438)  21.10 ± 0.14 | (n = 158)  20.60 ± 0.41 | (n = 126)  20.57 ± 0.46 | (n = 32)  20.71 ± 0.94 |
| Ordinal  x $\in$ {0, 0.5, 1}  0: Governing officials prohibit this activity.  0.5: Governing officials partially restrict this activity.  1: Governing officials allow this activity. | Breeding_  facilities_  numeric | (n = 14,380)  0.78 ± 0.00 | Not included | | | |
|  | Hunting_  enclosures_  numeric | (n = 14,380)  0.80 ± 0.00 | (n = 1,438)  0.80 ± 0.01 | (n = 158)  0.81 ± 0.03 | (n = 126)  0.81 ± 0.03 | (n = 32)  0.83 ± 0.06 |
|  | Interstate_  import_  of_live_  cervids_  numeric | (n = 14,380)  0.31 ± 0.00 | Not included | | | |
|  | Intrastate_  movement_  of_live_  cervids_  numeric | (n = 12,040)  0.49 ± 0.00 | Not included | | | |
|  | Baiting_  numeric | (n = 14,380)  0.38 ± 0.00 | (n = 1,438)  0.36 ± 0.01 | (n = 158)  0.35 ± 0.02 | (n = 126)  0.37 ± 0.03 | (n = 32)  0.31 ± 0.05 |
|  | Feeding_  numeric | (n = 14,380)  0.72 ± 0.00 | (n = 1,438)  0.69 ± 0.01 | (n = 158)  0.63 ± 0.02 | (n = 126)  0.62 ± 0.02 | (n = 32)  0.66 ± 0.05 |
|  | Whole_  carcass_  importation  _numeric | (n = 14,380)  0.41 ± 0.00 | (n = 1,438)  0.24 ± 0.01 | (n = 158)  0.22 ± 0.02 | (n = 126)  0.21 ± 0.02 | (n = 32)  0.27 ± 0.04 |
|  | Urine_  lures_  numeric | (n = 14,380)  0.89 ± 0.00 | (n = 1,438)  0.73 ± 0.01 | (n = 158)  0.69 ± 0.03 | (n = 126)  0.68 ± 0.04 | (n = 32)  0.72 ± 0.07 |
| Binary  x $\in$ {0, 1}  0: CWD has never been confirmed.  1: CWD has been confirmed. | Captive_  status | (n = 31,636)  0.00 ± 0.00 | (n = 1,438)  0.02 ± 0.00 | (n = 158)  0.05 ± 0.02 | (n = 126)  0.05 ± 0.02 | (n = 32)  0.06 ± 0.04 |

Table S2. Comparisons in accuracy, F1-score, precision, recall, and ROC-AUC among machine learning-based models for predicting chronic wasting disease (CWD) in wild white-tailed deer. Models include the Random Forest, Decision Tree, Gradient Boosting, and Light Gradient Boosting Classifier. Accuracy measures the proportion of correct predictions; F1 Score measures consistency; Precision measures correctness of positive predictions; Recall measures ability to identify true positives; and ROC measures the ratio between the False Positive Rate (the proportion of times the algorithm incorrectly predicted a CWD-positive) and the True Positive Rate (the proportion of times the algorithm correctly predicted a CWD-positive). The number in parentheses represents the average after the 5-fold validation.

| Algorithm | Accuracy | F1 Score | Precision | Recall | ROC |
| --- | --- | --- | --- | --- | --- |
| Random Forest | 0.7500  (0.7314) | 0.6875 | 0.7333 | 0.6471 | 0.7961 |
| Decision Tree | 0.7500  (0.6742) | 0.7879 | 0.8125 | 0.7647 | 0.8510 |
| Gradient Boosting Classifier | 0.8125  (0.7551) | 0.7879 | 0.8125 | 0.7647 | 0.8235 |
| Light Gradient Boosting Classifier | 0.7188  (0.7625) | 0.6875 | 0.7333 | 0.6471 | 0.7882 |

Table S3. Hyperparameters used in the machine learning-based models for predicting chronic wasting disease (CWD) in wild white-tailed deer. Models include Random Forest, Decision Tree, Gradient Boosting, and Light Gradient Boosting algorithms.

| Algorithm | Hyperparameters | | | | | | | |
| --- | --- | --- | --- | --- | --- | --- | --- | --- |
|  | N_estimators | Min_samples_split | Max_features | Max_depth | Min_samples_leaf | Min_child_samples | Learning_rate | Num_leaves |
| Random Forest | None | 2 | sqrt | None | 1 | --- | --- | --- |
| Decision Tree | --- | 5 | Log2 | 20 | 4 | --- | --- | --- |
| Gradient Boosting | 200 | 2 | Log2 | 3 | 1 | --- | 1 | --- |
| Light Gradient Boosting | 20 | --- | --- | 3 | --- | 40 | 1 | 31 |

Table S4. Sensitivity of predictions to changes in hunter harvest in the US state of Minnesota given *post hoc* proportional spatial allocation of harvest numbers from the original spatial unit (deer permit areas; DPAs) into counties. Predictions from this *CWD Prediction Web App* used the Light Gradient Boosting algorithm.

| Management Area | *CWD Prediction Web App* prediction | County-Approximation  (Harvest) | *CWD Prediction Web App* prediction is CWD-non detect when harvest is at or below | *CWD Prediction Web App* prediction is CWD-positive when harvest is at or above |
| --- | --- | --- | --- | --- |
| Aitkin | Positive | 5259 | 2900 | 2901 |
| Anoka | Negative | 1994 | 2003 | 2004 |
| Becker | Positive | 5201 | 2900 | 2901 |
| Beltrami | Positive | 3890 | 2900 | 2901 |
| Benton | Positive | 2941 | 1379 | 1380 |
| Big Stone | Negative | 597 | 2003 | 2004 |
| Blue Earth | Negative | 1106 | 1379 | 1380 |
| Brown | Negative | 792 | 1379 | 1380 |
| Carlton | Negative | 2403 | 2900 | 2901 |
| Carver | Negative | 683 | 1379 | 1380 |
| Cass | Positive | 6464 | 2900 | 2901 |
| Chippewa | Negative | 519 | 1379 | 1380 |
| Chisago | Positive | 2924 | 1379 | 1380 |
| Clay | Negative | 1090 | 1379 | 1380 |
| Clearwater | Negative | 2193 | 2900 | 2901 |
| Cook | Negative | 259 | 2900 | 2901 |
| Cottonwood | Negative | 632 | 2003 | 2004 |
| Crow Wing | Positive | 5693 | 2900 | 2901 |
| Dakota | Positive | 1606 | Always predicted CWD-positive. | |
| Dodge | Negative | 974 | 1379 | 1380 |
| Douglas | Positive | 3749 | 1379 | 1380 |
| Faribault | Negative | 576 | 2003 | 2004 |
| Fillmore | Positive | 3713 | Always predicted CWD-positive. | |
| Freeborn | Negative | 985 | 2003 | 2004 |
| Goodhue | Positive | 3475 | 1379 | 1380 |
| Grant | Negative | 666 | 2003 | 2004 |
| Hennepin | Negative | 1109 | 1379 | 1380 |
| Houston | Positive | 4528 | 1379 | 1380 |
| Hubbard | Positive | 4024 | 2900 | 2901 |
| Isanti | Positive | 3478 | 1379 | 1380 |
| Itasca | Positive | 4244 | 2900 | 2901 |
| Jackson | Negative | 541 | 1379 | 1380 |
| Kanabec | Positive | 3067 | 2900 | 2901 |
| Kandiyohi | Positive | 2366 | 2003 | 2004 |
| Kittson | Negative | 1452 | 2003 | 2004 |
| Koochiching | Negative | 2224 | 2900 | 2901 |
| Lac qui Parle | Negative | 906 | 1379 | 1380 |
| Lake | Negative | 546 | 2900 | 2901 |
| Lake of the Woods | Negative | 1425 | 2900 | 2901 |
| Le Sueur | Negative | 1127 | 2003 | 2004 |
| Lincoln | Negative | 606 | 2003 | 2004 |
| Lyon | Negative | 790 | 1379 | 1380 |
| Mahnomen | Negative | 524 | 2003 | 2004 |
| Marshall | Positive | 2817 | 2003 | 2004 |
| Martin | Negative | 568 | 2003 | 2004 |
| McLeod | Negative | 569 | 1379 | 1380 |
| Meeker | Negative | 1894 | 2003 | 2004 |
| Mille Lacs | Positive | 3997 | 2900 | 2901 |
| Morrison | Positive | 7424 | 2900 | 2901 |
| Mower | Negative | 1054 | 2003 | 2004 |
| Murray | Negative | 644 | 2003 | 2004 |
| Nicollet | Negative | 774 | 1379 | 1380 |
| Nobles | Negative | 417 | 2003 | 2004 |
| Norman | Positive | 1409 | 1379 | 1380 |
| Olmsted | Positive | 2794 | 1379 | 1380 |
| Otter Tail | Positive | 11662 | 1379 | 1380 |
| Pennington | Negative | 1214 | 1379 | 1380 |
| Pine | Positive | 5747 | 2900 | 2901 |
| Pipestone | Negative | 379 | 2003 | 2004 |
| Polk | Positive | 3241 | 2003 | 2004 |
| Pope | Positive | 2209 | 1379 | 1380 |
| Ramsey | Negative | 310 | 1379 | 1380 |
| Red Lake | Negative | 909 | 1379 | 1380 |
| Redwood | Negative | 714 | 2003 | 2004 |
| Renville | Negative | 1244 | 2003 | 2004 |
| Rice | Negative | 1469 | 2003 | 2004 |
| Rock | Negative | 253 | 1379 | 1380 |
| Roseau | Positive | 3054 | 2900 | 2901 |
| Scott | Negative | 974 | 1379 | 1380 |
| Sherburne | Positive | 2909 | 1379 | 1380 |
| Sibley | Negative | 657 | 1379 | 1380 |
| St. Louis | Positive | 6628 | 2900 | 2901 |
| Stearns | Positive | 6483 | 1379 | 1380 |
| Steele | Negative | 757 | 2003 | 2004 |
| Stevens | Negative | 535 | 2003 | 2004 |
| Swift | Negative | 938 | 1379 | 1380 |
| Todd | Positive | 7978 | 1379 | 1380 |
| Traverse | Negative | 488 | 2003 | 2004 |
| Wabasha | Positive | 3025 | 1379 | 1380 |
| Wadena | Positive | 3540 | 1379 | 1380 |
| Waseca | Negative | 575 | 2003 | 2004 |
| Washington | Negative | 1318 | 1379 | 1380 |
| Watonwan | Negative | 400 | 2003 | 2004 |
| Wilkin | Negative | 511 | 2003 | 2004 |
| Winona | Positive | 4065 | 1379 | 1380 |
| Wright | Positive | 2435 | 1379 | 1380 |
| Yellow Medicine | Negative | 788 | 1379 | 1380 |
